# Supplementary material for: Histopathological features of prostate cancer conspicuity on multiparametric MRI: protocol for a systematic review and meta-analysis
Source: BMJ Open. 2020 Oct 22;10(10):e039735. doi: 10.1136/bmjopen-2020-039735 (PMC7583062; doi:10.1136/bmjopen-2020-039735)
Supplement: Author's manuscript [file bmjopen-2020-039735.draft_revisions.pdf]

BMJ Open

BMJ Open is committed to open peer review. As part of this commitment we make the peer review history of every article we publish publicly available.

When an article is published we post the peer reviewers' comments and the authors' responses online. We also post the versions of the paper that were used during peer review. These are the versions that the peer review comments apply to.

The versions of the paper that follow are the versions that were submitted during the peer review process. They are not the versions of record or the final published versions. They should not be cited or distributed as the published version of this manuscript.

BMJ Open is an open access journal and the full, final, typeset and author-corrected version of record of the manuscript is available on our site with no access controls, subscription charges or pay-per-view fees (http://bmjopen.bmj.com).

If you have any questions on BMJ Open's open peer review process please email info.bmjopen@bmj.com

BMJ Open

Histopathological features of prostate cancer conspicuity on multiparametric MRI: a protocol for a systematic review and meta-analysis

Journal:	*BMJ Open*
Manuscript ID	bmjopen-2020-039735
Article Type:	Protocol
Date Submitted by the Author:	24-Apr-2020

Complete List of Authors:	Norris, Joseph; University College London, UCL Division of Surgery & Interventional Science<br>Carmona Echeverria, Lina; University College London, UCL Division of Surgery & Interventional Science<br>Simpson, Benjamin Scott; University College London, UCL Division of Surgery & Interventional Science<br>Ball, Rhys; University College London Hospitals NHS Foundation Trust, Department of Pathology<br>Freeman, Alex; University College London Hospitals NHS Foundation Trust, Department of Pathology<br>Kelly, Daniel; Cardiff University, School of Healthcare Sciences, College of Biomedical and Life Sciences<br>Kirkham, Alex; University College London Hospitals NHS Foundation Trust, Department of Radiology<br>Whitaker, Hayley; University College London, UCL Division of Surgery and Interventional Science<br>Emberton, Mark; University College London, UCL Division of Surgery & Interventional Sciences

Keywords:	Prostate disease < UROLOGY, Magnetic resonance imaging < RADIOLOGY & IMAGING, Histopathology < PATHOLOGY, Urological tumours < ONCOLOGY

SCHOLARONE™
Manuscripts

I, the Submitting Author has the right to grant and does grant on behalf of all authors of the Work (as defined in the below author licence), an exclusive licence and/or a non-exclusive licence for contributions from authors who are: i) UK Crown employees; ii) where BMJ has agreed a CC-BY licence shall apply, and/or iii) in accordance with the terms applicable for US Federal Government officers or employees acting as part of their official duties; on a worldwide, perpetual, irrevocable, royalty-free basis to BMJ Publishing Group Ltd ("BMJ") its licensees and where the relevant Journal is co-owned by BMJ to the co-owners of the Journal, to publish the Work in this journal and any other BMJ products and to exploit all rights, as set out in our licence.

The Submitting Author accepts and understands that any supply made under these terms is made by BMJ to the Submitting Author unless you are acting as an employee on behalf of your employer or a postgraduate student of an affiliated institution which is paying any applicable article publishing charge ("APC") for Open Access articles. Where the Submitting Author wishes to make the Work available on an Open Access basis (and intends to pay the relevant APC), the terms of reuse of such Open Access shall be governed by a Creative Commons licence – details of these licences and which Creative Commons licence will apply to this Work are set out in our licence referred to above.

Other than as permitted in any relevant BMJ Author's Self Archiving Policies, I confirm this Work has not been accepted for publication elsewhere, is not being considered for publication elsewhere and does not duplicate material already published. I confirm all authors consent to publication of this Work and authorise the granting of this licence.

For peer review only

Histopathological features of prostate cancer conspicuity on multiparametric MRI: a protocol for a systematic review and meta-analysis

Joseph M Norris,[1,2,3]* Lina M Carmona Echeverria,[1,3]* Benjamin S Simpson,[1] Rhys Ball,[5] Alex Freeman,[5] Daniel Kelly,[6] Alex Kirkham,[4] Hayley C Whitaker,[1] Mark Emberton[1,2]

*joint first-name authors

[1] UCL Division of Surgery & Interventional Science, University College London, London, UK

[2] London Deanery of Urology, London, UK

[3] Department of Urology, University College London Hospitals NHS Foundation Trust, London, UK

[4] Department of Radiology, University College London Hospitals NHS Foundation Trust, London, UK

[5] Department of Pathology, University College London Hospitals NHS Foundation Trust, London, UK

[6] School of Healthcare Sciences, Cardiff University, Wales, UK

Correspondence: Mr Joseph M Norris BSc (Hons), MSc, BM BS, MRCS (Eng)

Email: joseph.norris@ucl.ac.uk Phone: +44 7724022171 ORCID: 0000-0003-2294-0303

UCL Division of Surgery & Interventional Science, Charles Bell House, Foley Street, London, UK, W1W 7TS.

Article type: Systematic review protocol. Word count: 1,887 Ethics: Not required.

Keywords: Histopathology, multiparametric MRI, prostate cancer, systematic review.

Abbreviations: CD31, cluster of differentiation 31; DFFITS, difference in fits values; LOO, leave-one-out analyses; mpMRI, multiparametric magnetic resonance imaging; NICE, National Institute for Health and Care Excellence; *p*, probability value; PI-RADS, Prostate Imaging Reporting and Data System; PRISMA, Preferred Reporting Items for Systematic Reviews and Meta-Analyses; VEGF, Vascular Endothelial Growth Factor.

Funding: Norris is funded by the Medical Research Council (MRC) (Grant Reference: MR/S00680X/1). Carmona Echeverria is funded by a Prostate Cancer UK (PCUK) Centre of Excellence grant. Simpson is funded by the Rosetrees Trust.

Conflicts of Interest: Norris receives funding from the MRC. Simpson receives funding from the Rosetrees Trust. Carmona Echeverria receives funding from PCUK. Whitaker receives funding from PCUK, the Urology Foundation and the Rosetrees Trust. Kirkham, Freeman and Emberton have stock interest in Nuada Medical Ltd. Emberton receives funding from NIHR-i4i, MRC, Sonacare Inc., Trod Medical, Cancer Vaccine Institute and Sophiris Biocorp for trials in prostate cancer. Emberton is a medical consultant to Sonacare Inc., Sophiris Biocorp, Steba Biotech, GSK, Exact Imaging and Profound Medical. Travel allowance was previously provided from Sanofi Aventis, Astellas, GSK and Sonacare. Emberton is a proctor for HIFU with Sonacare Inc. and paid for training other surgeons in this procedure.

For peer review only - http://bmjopen.bmj.com/site/about/guidelines.xhtml

ABSTRACT

Introduction Multiparametric magnetic resonance imaging (mpMRI) has greatly improved the risk stratification and diagnostic approach for men with suspected clinically significant prostate cancer, due to the high proportion of significant disease that is detected. Importantly, mpMRI-visible tumours tend to be larger and of higher pathological grade than mpMRI-invisible tumours. However, concern remains around prostate cancer that is undetected by mpMRI and as such, there has been considerable recent research interest to investigate whether tumour conspicuity on mpMRI is associated with additional histopathological features (including, cellular density, microvessel density and unusual prostate cancer-subtypes) which may have important clinical implications in both diagnosis and prognosis. Furthermore, analysis of these features may help reveal the radiobiology that underpins the actual mechanisms of mpMRI-visibility (and invisibility) of prostate tumours. Here, we describe a protocol for a systematic review of the histopathological basis of prostate cancer conspicuity on mpMRI.

Methods and analysis A systematic search of the MEDLINE, PubMed, EMBASE and Cochrane databases will be conducted. The Preferred Reporting Items for Systematic Reviews and Meta-Analyses (PRISMA) guidelines will be used to guide screening, thematic reporting and conclusions drawn from all eligible studies. Included papers will be full text, English-language articles, comparing the histopathological characteristics of mpMRI-visible lesions and mpMRI-invisible tumours. Studies using confirmatory immunohistochemistry for the identification of immune subsets or structural components will be included. Study bias and quality will be assessed using a modified Newcastle-Ottawa scale. To ensure methodological rigour, this protocol is written in accordance with the PRISMA Protocol 2015 checklist. If appropriate, a meta-analysis will be conducted comparing histopathological feature frequency between mpMRI-visible and invisible disease.

Ethics and dissemination No ethical approval will be required, as this is an academic review of published literature. Findings will be disseminated through publications in peer-reviewed journals and presentations at national and international conferences.

Strengths and limitations of this study

► This work will represent the first detailed systematic review and meta-analysis of the histopathological features of mpMRI-visible and mpMRI-invisible prostate cancers, following the methodological steps of the PRISMA guidelines.

► The level of heterogeneity found between the reviewed articles somewhat limits the generalisability of derived results.

► The evidence surrounding important histopathological features of mpMRI-visible and mpMRI-invisible tumours is growing, however, as this is still a new area of research, the strength of the conclusions may be limited by finite extant literature.

For peer review only

BACKGROUND

The introduction of multiparametric magnetic resonance imaging (mpMRI) has greatly improved the approach to prostate cancer diagnosis, offering improved pre-biopsy risk stratification.[1] Accurate identification of high risk disease, before a biopsy is conducted, enables increased detection of significant cancer, and reduced detection of insignificant cancer, compared to traditional diagnostic approaches.[2] However, approximately 10-20% of clinically significant disease may be overlooked by mpMRI.[1,3] The mechanisms that underpin mpMRI-invisibility and the clinical implications that arise from this phenomenon have been the subject of intense research in recent years.

Prostate cancer visibility on mpMRI is positively associated with both tumour size and Gleason grade[1,4] which suggests tumour conspicuity can provide useful prognostication, as these pathological features at biopsy are typically considered to have the strongest impact on clinical outcome. Furthermore, it appears that this is corroborated at the molecular level, as mpMRI-visible tumours are enriched with genomic features of disease aggressivity.[5,6] More recently, nuanced microstructural and pathological features have also been linked with disease conspicuity including: cellular and microvessel density, cribriform and intraductal cancer patterns, and stromal and luminal (to malignant cell) ratios.[7-11] It is pertinent to now draw together this expanding radiopathological literature to address this important clinical challenge.

The aim of this systematic review is to appraise and collate, for the first time, the evidence supporting the histopathological basis of tumour visibility and invisibility on mpMRI, in order to reveal the potential mechanisms that underpin mpMRI conspicuity, and the possible diagnostic and prognostic implication of mpMRI phenotypes.

METHODS AND ANALYSIS

This systematic review protocol has been written in-line with the Preferred Reporting Items for Systematic Reviews and Meta-Analyses Protocol (PRISMA-P) 2015 checklist.[12] Once identified, included studies will be undergo analysis and thematic synthesis to derive the key histopathological elements associated with tumour visibility and invisibility on mpMRI.

Search methodology

The MEDLINE, PubMed, EMBASE and Cochrane databases will be systematically searched in order to retrieve all studies that contribute relevant evidence. The search strategy will include Medical Subject Headings (MESH) terms as well as free text, joined with appropriate Boolean operators. The search will include the terms: ‘prostate,’ ‘cancer’ and ‘MRI,’ as well as multiple synonyms for ‘pathology’ and ‘histology’. The selected search terms are broad, with high sensitivity for identification of relevant studies. We will expedite the systematic review process by uploading all articles to Rayyan, a semi-automated tool designed to improve the speed and reporting accuracy during the initial screening process and to allow three reviewers to filter duplicate studies and screen articles for relevance.[13] To further improve the evidence yield, all included articles will be reference-searched manually to identify missed studies or additional data. Finally, experts will be consulted to identify additional literature. In the case of missing or unclear data, the corresponding authors will be contacted directly.

Study selection and data extraction

Three researchers will independently screen eligible studies, removing irrelevant studies based on titles and abstracts. Those studies which pass the initial screen will be downloaded and the full-text examined to confirm eligibility. Disagreement between reviewers will be discussed until a consensus is reached or a fourth reviewer will be consulted. All exclusions will be noted for later analysis and the reasons for exclusion documented in detail in order to generate the PRISMA flow diagram.

Inclusion criteria

To be included in the analysis, studies must investigate one or more histopathological aspects of the appearance of prostate cancer on mpMRI. Investigations may be conducted at the macroscopic-level (for example, tumour size); at microscopic-level (for example, morphological and pathological patterns); or be based upon routine or special staining, including immunohistochemistry (for example, confirmation of microstructural components, such as CD31 for microvessels).

Exclusion criteria

Non-English language articles, conference abstracts, review articles, correspondence articles, expert opinions and case reports will be excluded. Studies that do not correlate mpMRI phenotypes with histopathological features will be excluded. Articles focusing solely on molecular characteristics (genetic or transcriptomic) or clinical features of mpMRI conspicuity will also be removed.

Data extraction

All relevant articles will be carefully read and themes extracted. All extracted data will be held on a shared datasheet and confirmed by at least three independent reviewers to maintain veracity. Data will be collated in a manner that we have successfully demonstrated previously.[6] Table 1 summarises data items to be collected.

For peer review only

Table 1 Data collection items

Item no.	Data title	Data type
1	Year of publication	Study characteristic
2	Study authors	Study characteristic
3	Experimental design	Study characteristic
4	Patient population	Demographics
5	Study size	Demographics
6	mpMRI scoring scheme used	Methodology
7	Definition for clinically significant disease	Methodology
8	Definition for lesion visibility & invisibility	Methodology
9	Sample processing approach	Methodology
10	Histopathological feature studied	Outcome
11	Differential quantification of feature	Outcome

mpMRI, multiparametric magnetic resonance imaging; no., number.

Endpoints

The primary endpoint will be statistically significant differences in quantitative measurements (for example, frequency or density) of histopathological features between mpMRI-visible and mpMRI-invisible prostate cancers. Secondary endpoints will include explanatory links between these features and mpMRI-conspicuity, as well as the potential clinical implications.

Risk of bias in individual studies

A modified Newcastle-Ottawa score (constructed for assessment of observational cohort studies) will be used to score the bias and quality across included studies.[14] This system is divided into three core elements: selection, comparability and outcome. Within each section, there are sub-questions to evaluate the quality of the research methodology, at the study level. Three reviewers will be involved with this process and any disagreement will be settled by consensus. The outcome of the bias and quality assessment will inform the thematic synthesis by providing an assessment of the reliability and applicability of the available evidence. If studies are deemed to be of excessively low quality (or high bias) then these may be excluded, or if included, will be accompanied by appropriate commentary in the discussion and conclusion. As this review is focused on the histopathological characteristics associated with prostate cancer conspicuity on mpMRI (as opposed to treatment outcome), we will modify non-applicable sections of the scoring scheme to more closely reflect the nature of the evidence base and reduce reporting inaccuracy, in a similar approach taken previously.[6]

For peer review only

Meta-analysis

If there are a sufficient number of studies available (i.e. over three) that analyse a particular histopathological feature using a similar methodology, then we will conduct a meta-analysis. The frequency of the histopathological feature between mpMRI-visible and mpMRI-invisible tumours will be compared. Any analysis will be performed as previously described.[15] Briefly, numbers of positive and negative cases of a particular histopathological feature would be extracted from a given study and raw/direct proportions will be calculated. The distribution of untransformed, logit and double arcsine transformed proportions will be compared. Whichever distributions resemble a normal distribution (assessed using density plots and Shapiro-Wilk tests) will be used for further analysis.

The model fitted will be determined based on inter-study variation (measured via I^2); if significant, a random-effects model will be fitted, or a fixed-effect model, if not. After fitting a model to all relevant studies, leave-one-out analyses (LOO) and accompanying diagnostic plots will be used to identify influential studies including: externally studentised residuals, difference in fits values (DFFITS), Cook's distances, covariance ratios, LOO estimates of the amount of heterogeneity, LOO values of the test statistics for heterogeneity, hat values and weights. Studies with a statistically significant influence on the fitted model will be removed as outliers and the model re-fitted. Finally, the summary estimates will be compared between mpMRI-visible and mpMRI-invisible cancers as subgroups, and the significance of differences will be assessed. If appropriate, moderator analysis will also be performed between factors which may influence the outcome, including, but not limited to: study size, year of publication, cohort type, relevant methodology or visibility definition. Analytics will be performed as outlined by Wang.[16]

For peer review only

DISCUSSION

Over the past decade, mpMRI has been widely adopted as the risk stratification tool-of-choice for men at risk of prostate cancer. This has become particularly true following the incorporation of pre-biopsy mpMRI into national and international prostate cancer guidelines.[17,18] As such, it is now crucial that we better appreciate the characteristics of prostate cancers that are detected and missed by mpMRI.[4] Through systematic review, we aim to identify commonality between pathology-based studies which have investigated mpMRI-visible and mpMRI-invisible tumours. Our results will enhance the understanding of the histopathological features that influence prostate cancer visibility on mpMRI, particularly beyond increased Gleason grade and high tumour volume.

Estimates for proportions of significant mpMRI-undetected prostate cancer vary between studies,[19] however, it has become clear that microscopic tumour composition is likely an important factor in determining mpMRI signal (and therefore disease detection). Our planned thematic synthesis will assimilate the evidence surrounding the microscopic basis of tumour conspicuity, including the following considerations. Diffusion of water is a key principle of MRI signal generation; this is affected by alterations in tissue microenvironment and cellular structure, which may hamper free-movement of water molecules.[20] This concept theoretically explains the disparate MRI appearances of the different zones of the prostate (including, the peripheral zone and normal central gland) and is also likely to be crucial in the mpMRI-visibility of prostate cancer.[21] Indeed, an increased architectural density has been observed in mpMRI-visible tumours, with an increased proportion of cancer cells and decreased proportions of stroma and luminal spaces.[22] This may, in turn, explain visibility of these tumours on mpMRI, through increased restriction of water diffusion within dense tumour tissue (thus generating high signal on the diffusion weighted mpMRI sequence). Furthermore, it appears that mpMRI-visible tumours have raised microvessel density,[23] which is cohesive with genetic studies that also have demonstrated an enrichment of Vascular Endothelial Growth Factor (VEGF) in these tumours.[24,25] An increased level of vasculature could potentially explain tumour visibility on mpMRI, through increased concentrations of mpMRI-contrast agent (gadolinium) in tumour vessels, thus generating higher MRI signal on the dynamic contrast sequence. Finally, there is mixed evidence regarding the mpMRI-visibility or the mpMRI-invisibility of particular prostate cancer subtypes, including cribriform pattern[26,27] and ductal cancer.[28] This is potentially concerning, as these cancer subtypes are associated with increased disease aggression and poor clinical outcome. Our planned systematic review (described in this protocol) will highlight and discuss these postulated features, among many others, in detail.

In summary, this systematic review will combine the extant evidence in this emerging field, for the first time. Collation and analysis of this data will enrich our understanding of the additional histopathological factors (beyond tumour grade and size) that contribute to the conspicuity of prostate cancer on mpMRI. Additionally, this process will also help reveal the potential clinical role that these factors play, and will aid identification of important avenues for future research.

For peer review only - http://bmjopen.bmj.com/site/about/guidelines.xhtml

Trial status

► Preliminary searches: started.

► Piloting of the study selection process: started.

► Formal screening: started.

► Data extraction: not started.

► Risk of bias assessment: not started.

► Data analysis: not started.

Draft of search strategy for MEDLINE, EMBASE, PubMed and Cochrane Databases:

((((prostat* NOT prostatitis) AND ("cancer" OR tumo?r* OR malignancy*)) AND ("magnetic resonance imaging" OR "MRI" OR "multiparametric magnetic resonance imaging" OR "mpMRI" OR "mp-MRI")) AND (patholog* OR histopatholog* OR histo* OR "IHC" OR "Immunohistochemistry")) AND visib*).ti,ab

ETHICS AND DISSEMINATION

Due to the nature of the study, there are no relevant ethical concerns and informed consent will not be required. The protocol and systematic review will be disseminated via a peer-reviewed journal.

Author affiliations

[1] UCL Division of Surgery & Interventional Science, University College London, London, UK

[2] London Deanery of Urology, London, UK

[3] Department of Urology, University College London Hospitals NHS Foundation Trust, London, UK

[4] Department of Radiology, University College London Hospitals NHS Foundation Trust, London, UK

[5] Department of Pathology, University College London Hospitals NHS Foundation Trust, London, UK

[6] School of Healthcare Sciences, Cardiff University, Wales, UK

Twitter Joseph M Norris @MrJosephNorris, Lina M Carmona Echeverria @linamacarmona, Benjamin S Simpson @b3nsimp, Alex Freeman @alexpathologist, Daniel Kelly @profdkelly, Hayley C Whitaker @HayleyWhitaker, Mark Emberton @EmbertonMark

Contributors The authors' contribution includes, but is not limited to, the following: JMN, LMCE and BSS drafted the manuscript and created the study concept. RB, AK, AF, DK, HCW and ME provided supervision and guidance during the study. All authors reviewed and approved the manuscript in its current form.

Funding JMN is funded by the Medical Research Council (MRC) (Grant Reference: MR/S00680X/1). LMCE receives funding from Prostate Cancer UK (PCUK). BSS receives funding from the Rosetrees Trust.

Competing interests Norris receives funding from the MRC. Simpson receives funding from the Rosetrees Trust. Carmona Echeverria receives funding from PCUK. Whitaker receives funding from PCUK, the Urology Foundation and the Rosetrees Trust. Kirkham, Freeman and Emberton

have stock interest in Nuada Medical Ltd. Emberton receives funding from NIHR-i4i, MRC, Sonacare Inc., Trod Medical, Cancer Vaccine Institute and Sophiris Biocorp for trials in prostate cancer. Emberton is a medical consultant to Sonacare Inc., Sophiris Biocorp, Steba Biotech, GSK, Exact Imaging and Profound Medical. Travel allowance was previously provided from Sanofi Aventis, Astellas, GSK and Sonacare. Emberton is a proctor for HIFU with Sonacare Inc. and paid for training other surgeons in this procedure.

Patient consent for publication Not required.

Provenance and peer review Not commissioned; externally peer reviewed.

Open access This is an open access article distributed in accordance with the Creative Commons Attribution 4.0 Unported (CC BY 4.0) license, which permits others to copy, redistribute, remix, transform and build upon this work for any purpose, provided the original work is properly cited, a link to the licence is given, and indication of whether changes were made. See: https://creativecommons.org/licenses/by/4.0/.

ORCID IDs

Joseph M Norris http://orcid.org/0000-0003-2294-0303

Benjamin S Simpson http://orcid.org/0000-0003-3685-6110

For peer review only

REFERENCES

1. Ahmed HU, El-Shater Bosaily A, Brown LC, *et al*. Diagnostic accuracy of multi-parametric MRI and TRUS biopsy in prostate cancer (PROMIS): a paired validating confirmatory study. *Lancet* 2017;389:815–22.
2. Kasivisvanathan V, Rannikko AS, Borghi M, *et al*. MRI-targeted or standard biopsy for prostate-cancer diagnosis. *N Engl J Med* 2018;378:1767–77.
3. Radtke JP, Kuru TH, Boxler S, *et al*. Comparative analysis of transperineal template saturation prostate biopsy versus magnetic resonance imaging targeted biopsy with magnetic resonance imaging-ultrasound fusion guidance. *J Urol* 2015;193:87–94.
4. Norris JM, Carmona Echeverria LM, Bott SRJ, *et al.* Which prostate cancers are overlooked by mpMRI? An analysis from PROMIS. *J Urol* 2020;203:e1243–4.
5. Norris JM, Simpson BS, Parry MA, *et al*. Genetic correlates of prostate cancer visibility (and invisibility) on mpMRI: it's time to take stock. *BJU Int* 2020;125:340–2.
6. Norris JM, Simpson, BS, Parry MA, *et al*. Genetic landscape of prostate cancer conspicuity on multiparametric MRI: a protocol for a systematic review and bioinformatic analysis. *BMJ Open* 2020;10:e03461.
7. Bill-Axelson A, Holmberg L, Garmo H, *et al*. Radical prostatectomy or watchful waiting in prostate cancer – 29-year follow-up. *NEJM* 2018;379:2319–29.
8. Kir G, Sarbay BC, Gümüş E, *et al*. The association of the cribriform pattern with outcome for prostatic adenocarcinomas. *Pathol Res Pract* 2014;210:640–4.
9. Kweldam CF, Wildhagen MF, Steyerberg EW, *et al*. Cribriform growth is highly predictive for postoperative metastasis and disease-specific death in Gleason score 7 prostate cancer. *Mod Pathol* 2015;28:457–64.
10. Khani F, Epstein JI. Prostate biopsy specimens with Gleason 3+3=6 and intraductal carcinoma. *Am J Surg Pathol* 2015;39:1383–9.
11. Mucci LA, Powolny A, Giovannucci E, *et al*. Prospective study of prostate tumor angiogenesis and cancer-specific mortality in the health professionals follow-up study. *J Clin Oncol* 2009;27:5627–33.
12. Moher D, Shamseer L, Clarke M, *et al*. Preferred reporting items for systematic review and meta-analysis protocols (PRISMA-P) 2015 statement. *Syst Rev* 2015;4:1.
13. Wells G, Shea B, Oconnell D. The Newcastle-Ottawa scale (NOS) for assessing the quality of nonrandomized studies in meta-analyses. Available from: http://www.ohri.ca/programs/clinical_epidemiology/oxford.asp
14. Ouzzani M, Hammady H, Fedorowicz Z, *et al*. Rayyan—a web and mobile app for systematic reviews. *Syst Rev* 2016 Dec;5(1):210.
15. O'Connor E, Simpson BS, Houlden H, *et al*. Prevalence of familial cluster headache: a systematic review and meta-analysis. *J Headache Pain* 2020: In Press.
16. Wang, N. How to conduct a meta-analysis of proportions in R: a comprehensive tutorial. Available from DOI: 10.13140/RG.2.2.27199.00161.
17. Wise J. NICE recommends MRI for suspected prostate cancer to reduce biopsies. BMJ 2018;363:k5290.
18. Dasgupta P, Davis J, Hughes S. NICE guidelines on prostate cancer 2019. *BJU Int* 2019;124:1.
19. Le JD, Tan N, Shkolyar E, *et al*. Multifocality and prostate cancer detection by multiparametric magnetic resonance imaging: correlation with whole-mount histopathology. *Eur Urol* 2015;67:569–76.
20. Le Bihan D. Molecular diffusion nuclear magnetic resonance imaging. *Magn Reson Q* 1991;7:1–30.

For peer review only

21. Kozlowski P, Chang SD, Jones EC, *et al*. Combined diffusion-weighted and dynamic contrast-enhanced MRI for prostate cancer diagnosis—correlation with biopsy and histopathology. *J Magn Reson Imaging* 2006;24:108–13.

22. Miyai K, Mikoshi A, Hamabe F, *et al*. Histological differences in cancer cells, stroma, and luminal spaces strongly correlate with in vivo MRI-detectability of prostate cancer. *Mod Pathol* 2019;32:1536–43.

23. Borren A, Groenendaal G, Moman MR, *et al*. Accurate prostate tumour detection with multiparametric magnetic resonance imaging: dependence on histological properties. *Acta Oncol* 2014;53:88–95.

24. Lenkinski RE, Bloch BN, Liu F, *et al*. Mapping MRI/MRS parameters with genetic over-expression profiles in human prostate cancer: demonstrating the potential. *MAGMA* 2008;21:411–21.

25. Li P, You S, Nguyen C, *et al*. Genes involved in prostate cancer progression determine MRI visibility. *Theranostics* 2018;8:1752–65.

26. Tonttila PP, Ahtikoski A, Kuisma M, *et al*. Multiparametric MRI prior to radical prostatectomy identifies intraductal and cribriform growth patterns in prostate cancer. *BJU Int* 2019;124:992–99.

27. Truong M, Feng C, Hollenberg G, *et al*. A comprehensive analysis of cribriform morphology on magnetic resonance imaging/ultrasound fusion biopsy correlated with radical prostatectomy specimens. *J Urol* 2018;199:106–13.

28. Schieda N, Coffey N, Gulavita P, *et al*. Prostatic ductal adenocarcinoma: an aggressive tumour variant unrecognized on T2 weighted magnetic resonance imaging (MRI). *Eur Radiol* 2014;24:1349–56.

For peer review only

PRISMA-P (Preferred Reporting Items for Systematic review and Meta-Analysis Protocols) 2015 checklist: recommended items to address in a systematic review protocol*

	Section and topic	Item No	Checklist item
	**ADMINISTRATIVE INFORMATION**		
	Title:		
✓ Page 1	Identification	1a	Identify the report as a protocol of a systematic review
✗ N/A	Update	1b	If the protocol is for an update of a previous systematic review, identify as such
✗ See cover letter	Registration	2	If registered, provide the name of the registry (such as PROSPERO) and registration number
	Authors:		
✓ Page 1	Contact	3a	Provide name, institutional affiliation, e-mail address of all protocol authors; provide physical mailing address of corresponding author
✓ Page 8	Contributions	3b	Describe contributions of protocol authors and identify the guarantor of the review
✗ N/A	Amendments	4	If the protocol represents an amendment of a previously completed or published protocol, identify as such and list changes; otherwise, state plan for documenting important protocol amendments
	Support:		
✓ Page 8	Sources	5a	Indicate sources of financial or other support for the review
✓ Page 8	Sponsor	5b	Provide name for the review funder and/or sponsor
✓ Page 8	Role of sponsor or funder	5c	Describe roles of funder(s), sponsor(s), and/or institution(s), if any, in developing the protocol
	**INTRODUCTION**		
✓ Page 3	Rationale	6	Describe the rationale for the review in the context of what is already known
✓ Page 5	Objectives	7	Provide an explicit statement of the question(s) the review will address with reference to participants, interventions, comparators, and outcomes (PICO)
	**METHODS**		
✓ Page 4	Eligibility criteria	8	Specify the study characteristics (such as PICO, study design, setting, time frame) and report characteristics (such as years considered, language, publication status) to be used as criteria for eligibility for the review
✓ Page 4	Information sources	9	Describe all intended information sources (such as electronic databases, contact with study authors, trial registers or other grey literature sources) with planned dates of coverage
✓ Page 8	Search strategy	10	Present draft of search strategy to be used for at least one electronic database, including planned limits, such that it could be repeated
	Study records:		
✓ Page 4/5	Data management	11a	Describe the mechanism(s) that will be used to manage records and data throughout the review

For peer review only

✓ Page 4	Selection process	11b	State the process that will be used for selecting studies (such as two independent reviewers) through each phase of the review (that is, screening, eligibility and inclusion in meta-analysis)
✓ Page 5	Data collection process	11c	Describe planned method of extracting data from reports (such as piloting forms, done independently, in duplicate), any processes for obtaining and confirming data from investigators
✓ Page 5	Data items	12	List and define all variables for which data will be sought (such as PICO items, funding sources), any pre-planned data assumptions and simplifications
✓ Page 5	Outcomes and prioritization	13	List and define all outcomes for which data will be sought, including prioritization of main and additional outcomes, with rationale
✓ Page 5	Risk of bias in individual studies	14	Describe anticipated methods for assessing risk of bias of individual studies, including whether this will be done at the outcome or study level, or both; state how this information will be used in data synthesis
✓ Page 6	Data synthesis	15a	Describe criteria under which study data will be quantitatively synthesised
		15b	If data are appropriate for quantitative synthesis, describe planned summary measures, methods of handling data and methods of combining data from studies, including any planned exploration of consistency (such as I^2, Kendall's τ)
		15c	Describe any proposed additional analyses (such as sensitivity or subgroup analyses, meta-regression)
		15d	If quantitative synthesis is not appropriate, describe the type of summary planned
✓ Page 5	Meta-bias(es)	16	Specify any planned assessment of meta-bias(es) (such as publication bias across studies, selective reporting within studies)
✓ Page 5	Confidence in cumulative evidence	17	Describe how the strength of the body of evidence will be assessed (such as GRADE)

*** It is strongly recommended that this checklist be read in conjunction with the PRISMA-P Explanation and Elaboration (cite when available) for important clarification on the items. Amendments to a review protocol should be tracked and dated. The copyright for PRISMA-P (including checklist) is held by the PRISMA-P Group and is distributed under a Creative Commons Attribution Licence 4.0.**

From: Shamseer L, Moher D, Clarke M, Ghersi D, Liberati A, Petticrew M, Shekelle P, Stewart L, PRISMA-P Group. Preferred reporting items for systematic review and meta-analysis protocols (PRISMA-P) 2015: elaboration and explanation. BMJ. 2015 Jan 2;349(jan02 1):g7647.

For peer review only

BMJ Open

Histopathological features of prostate cancer conspicuity on multiparametric MRI: a protocol for a systematic review and meta-analysis

Journal:	*BMJ Open*
Manuscript ID	bmjopen-2020-039735.R1
Article Type:	Protocol
Date Submitted by the Author:	24-Aug-2020
Complete List of Authors:	Norris, Joseph; University College London, UCL Division of Surgery & Interventional Science<br>Carmona Echeverria, Lina; University College London, UCL Division of Surgery & Interventional Science<br>Simpson, Benjamin Scott; University College London, UCL Division of Surgery & Interventional Science<br>Ball, Rhys; University College London Hospitals NHS Foundation Trust, Department of Pathology<br>Freeman, Alex; University College London Hospitals NHS Foundation Trust, Department of Pathology<br>Kelly, Daniel; Cardiff University, School of Healthcare Sciences, College of Biomedical and Life Sciences<br>Kirkham, Alex; University College London Hospitals NHS Foundation Trust, Department of Radiology<br>Whitaker, Hayley; University College London, UCL Division of Surgery and Interventional Science<br>Emberton, Mark; University College London, UCL Division of Surgery & Interventional Sciences
**Primary Subject Heading**:	Urology
Secondary Subject Heading:	Oncology, Radiology and imaging, Research methods, Evidence based practice, Pathology
Keywords:	Prostate disease < UROLOGY, Magnetic resonance imaging < RADIOLOGY & IMAGING, Histopathology < PATHOLOGY, Urological tumours < ONCOLOGY

SCHOLARONE™
Manuscripts

I, the Submitting Author has the right to grant and does grant on behalf of all authors of the Work (as defined in the below author licence), an exclusive licence and/or a non-exclusive licence for contributions from authors who are: i) UK Crown employees; ii) where BMJ has agreed a CC-BY licence shall apply, and/or iii) in accordance with the terms applicable for US Federal Government officers or employees acting as part of their official duties; on a worldwide, perpetual, irrevocable, royalty-free basis to BMJ Publishing Group Ltd ("BMJ") its licensees and where the relevant Journal is co-owned by BMJ to the co-owners of the Journal, to publish the Work in this journal and any other BMJ products and to exploit all rights, as set out in our licence.

The Submitting Author accepts and understands that any supply made under these terms is made by BMJ to the Submitting Author unless you are acting as an employee on behalf of your employer or a postgraduate student of an affiliated institution which is paying any applicable article publishing charge ("APC") for Open Access articles. Where the Submitting Author wishes to make the Work available on an Open Access basis (and intends to pay the relevant APC), the terms of reuse of such Open Access shall be governed by a Creative Commons licence – details of these licences and which Creative Commons licence will apply to this Work are set out in our licence referred to above.

Other than as permitted in any relevant BMJ Author's Self Archiving Policies, I confirm this Work has not been accepted for publication elsewhere, is not being considered for publication elsewhere and does not duplicate material already published. I confirm all authors consent to publication of this Work and authorise the granting of this licence.

For peer review only

Histopathological features of prostate cancer conspicuity on multiparametric MRI: a protocol for a systematic review and meta-analysis

Joseph M Norris,[1,2,3] Lina M Carmona Echeverria,[1,3]* Benjamin S Simpson,[1] Rhys Ball,[5] Alex Freeman,[5] Daniel Kelly,[6] Alex Kirkham,[4] Hayley C Whitaker,[1] Mark Emberton[1,2]

JMN and LMCE are joint first authors.

[1] UCL Division of Surgery & Interventional Science, University College London, London, UK

[2] London Deanery of Urology, London, UK

[3] Department of Urology, University College London Hospitals NHS Foundation Trust, London, UK

[4] Department of Radiology, University College London Hospitals NHS Foundation Trust, London, UK

[5] Department of Pathology, University College London Hospitals NHS Foundation Trust, London, UK

[6] School of Healthcare Sciences, Cardiff University, Wales, UK

Correspondence to: Joseph M Norris; joseph.norris@ucl.ac.uk

Article type: Systematic review protocol Word count: 1,778 Ethics: Not required

Funding: Norris is funded by the Medical Research Council (MRC) (MR/S00680X/1). Carmona Echeverria is funded by a Prostate Cancer UK (PCUK) Centre of Excellence grant. Simpson is funded by the Rosetrees Trust.

Conflicts of Interest: Norris receives funding from the MRC. Simpson receives funding from the Rosetrees Trust. Carmona Echeverria receives funding from PCUK. Whitaker receives funding from PCUK, the Urology Foundation and the Rosetrees Trust. Kirkham, Freeman and Emberton have stock interest in Nuada Medical Ltd. Emberton receives funding from NIHR-i4i, MRC, Sonacare Inc., Trod Medical, Cancer Vaccine Institute and Sophiris Biocorp for trials in prostate cancer. Emberton is a medical consultant to Sonacare Inc., Sophiris Biocorp, Steba Biotech, GSK, Exact Imaging and Profound Medical. Travel allowance was previously provided from Sanofi Aventis, Astellas, GSK and Sonacare. Emberton is a proctor for HIFU with Sonacare Inc. and is paid for training other surgeons in this procedure.

For peer review only

ABSTRACT

Introduction Multiparametric magnetic resonance imaging (mpMRI) has improved risk stratification for men with suspected prostate cancer. Indeed, mpMRI-visible tumours tend to be larger and of higher pathological grade than mpMRI-invisible tumours, however, concern remains around significant cancer that is undetected by mpMRI. There has been considerable recent interest to investigate whether tumour conspicuity on mpMRI is associated with additional histopathological features (including, cellular density, microvessel density and unusual prostate cancer-subtypes) which may have important clinical implications in both diagnosis and prognosis. Furthermore, analysis of these features may help reveal the radiobiology that underpins the actual mechanisms of mpMRI-visibility (and invisibility) of prostate tumours. Here, we describe a protocol for a systematic review of the histopathological basis of prostate cancer conspicuity on mpMRI.

Methods and analysis A systematic search of the MEDLINE, PubMed, EMBASE and Cochrane databases will be conducted. The Preferred Reporting Items for Systematic Reviews and Meta-Analyses (PRISMA) guidelines will be used to guide screening, thematic reporting and conclusions drawn from all eligible studies. Included papers will be full text, English-language articles, comparing the histopathological characteristics of mpMRI-visible lesions and mpMRI-invisible tumours. All studies published between January 1950 and January 2020 will be eligible for inclusion. Studies using confirmatory immunohistochemistry for the identification of immune subsets or structural components will be included. Study bias and quality will be assessed using a modified Newcastle-Ottawa scale. To ensure methodological rigour, this protocol is written in accordance with the PRISMA Protocol 2015 checklist. If appropriate, a meta-analysis will be conducted comparing histopathological feature frequency between mpMRI-visible and mpMRI-invisible disease.

Ethics and dissemination No ethical approval will be required, as this is an academic review of published literature. Findings will be disseminated through publications in peer-reviewed journals and presentations at national and international conferences. PROSPERO Registration Number: CRD42020176049.

Strengths and limitations of this study

► This work will represent the first detailed systematic review and meta-analysis of the histopathological features of mpMRI-visible and mpMRI-invisible prostate cancers, following the methodological steps of the PRISMA guidelines.

► The level of heterogeneity found between the reviewed articles somewhat limits the generalisability of derived results.

► The evidence surrounding important histopathological features of mpMRI-visible and mpMRI-invisible tumours is growing, however, as this is still a new area of research, the strength of the conclusions may be limited by finite extant literature.

For peer review only

BACKGROUND

The introduction of multiparametric magnetic resonance imaging (mpMRI) has greatly improved the approach to prostate cancer diagnosis, offering improved pre-biopsy risk stratification.[1] Accurate identification of high risk disease, before a biopsy is conducted, enables increased detection of significant cancer, and reduced detection of insignificant cancer, compared to traditional diagnostic approaches.[2] However, approximately 10-20% of clinically significant disease may be overlooked by mpMRI.[1,3] The mechanisms that underpin mpMRI-invisibility and the clinical implications that arise from this phenomenon have been the subject of intense research in recent years.

Prostate cancer visibility on mpMRI is positively associated with both tumour size and Gleason grade[1,4] which suggests tumour conspicuity can provide useful prognostication, as these pathological features at biopsy are typically considered to have the strongest impact on clinical outcome. Furthermore, it appears that this is corroborated at the molecular level, as mpMRI-visible tumours are enriched with genomic features of disease aggressivity.[5,6] More recently, nuanced microstructural and pathological features have also been linked with disease conspicuity including: cellular and microvessel density, cribriform and intraductal cancer patterns, and stromal and luminal (to malignant cell) ratios.[7-11] Taken together, this evidence suggests that the visibility status of a tumour on mpMRI may be an indicator of clinical risk. Early evidence has shown that low-risk Gleason Grade Group (GGG) 1 tumours with a positive mpMRI at baseline are at increased risk of intervention, upgrading, and unfavourable disease at the time of radical prostatectomy, compared to those with GGG1 mpMRI-invisible tumours.[12] However, mpMRI-targeted biopsy may not be insufficient alone to estimate risk of disease progression.[13] As the ability of mpMRI to detect tumours is influenced by additional histopathological features, it is pertinent to now draw together this expanding radiopathological literature to address this important clinical challenge.

The aim of this systematic review is to appraise and collate, for the first time, the evidence supporting the histopathological basis of tumour visibility and invisibility on mpMRI, in order to reveal the potential mechanisms that underpin mpMRI conspicuity, and the possible diagnostic and prognostic implication of mpMRI phenotypes.

For peer review only

METHODS AND ANALYSIS

This systematic review protocol has been written in-line with the Preferred Reporting Items for Systematic Reviews and Meta-Analyses Protocol (PRISMA-P) 2015 checklist.[14] Once identified, included studies will undergo analysis and thematic synthesis to derive the key histopathological elements associated with tumour visibility and invisibility on mpMRI. The protocol for this review is registered on PROSPERO under the registration number: CRD42020176049.

Search methodology

The MEDLINE, PubMed, EMBASE and Cochrane databases will be systematically searched in order to retrieve all studies that contribute relevant evidence. The search strategy will include Medical Subject Headings (MESH) terms as well as free text, joined with appropriate Boolean operators. The search will include the terms: 'prostate,' 'cancer' and 'MRI,' as well as multiple synonyms for the terms 'pathology' and 'histology'. The selected search terms are broad, with high sensitivity for identification of relevant studies. To include the maximum number of articles, all studies published between January 1950 and January 2020 will be eligible. We will expedite the systematic review process by uploading all articles to Rayyan, a semi-automated tool designed to improve the speed and reporting accuracy during the initial screening process and to allow three reviewers to filter duplicate studies and screen articles for relevance.[15] To further improve the evidence yield, all included articles will be reference-searched manually to identify missed studies or additional data. Finally, experts will be consulted to identify additional literature. In the case of missing or unclear data, the corresponding authors will be contacted directly.

Study selection and data extraction

Three researchers will independently screen eligible studies, removing irrelevant studies based on titles and abstracts. Those studies which pass the initial screen will be downloaded and the full-text examined to confirm eligibility. Disagreement between reviewers will be discussed until a consensus is reached or a fourth reviewer will be consulted. All exclusions will be noted for later analysis and the reasons for exclusion documented in detail in order to generate the PRISMA flow diagram.

Inclusion criteria

To be included in the analysis, studies must investigate one or more histopathological aspects of the appearance of prostate cancer on mpMRI. Investigations may be conducted at the macroscopic-level (for example, tumour size); at microscopic-level (for example, morphological and pathological patterns); or be based upon routine or special staining, including immunohistochemistry (for example, confirmation of microstructural components, such as CD31 for microvessels).

Exclusion criteria

Non-English language articles, conference abstracts, review articles, correspondence articles, expert opinions and case reports will be excluded. Studies that do not correlate mpMRI phenotypes with histopathological features will be excluded. Articles focusing solely on molecular characteristics (genetic or transcriptomic) or clinical features of mpMRI conspicuity will also be removed.

Data extraction

For peer review only - http://bmjopen.bmj.com/site/about/guidelines.xhtml

All relevant articles will be carefully read and themes extracted. All extracted data will be held on a shared datasheet and confirmed by at least three independent reviewers to maintain veracity. Data will be collated in a manner that we have successfully demonstrated previously.[6] Table 1 summarises data items to be collected.

Table 1 Data collection items

Item no.	Data title	Data type
1	Year of publication	Study characteristic
2	Study authors	Study characteristic
3	Experimental design	Study characteristic
4	Patient population	Demographics
5	Study size	Demographics
6	mpMRI scoring scheme used	Methodology
7	Definition for clinically significant disease	Methodology
8	Definition for lesion visibility & invisibility	Methodology
9	Sample processing approach	Methodology
10	Histopathological feature studied	Outcome
11	Differential quantification of feature	Outcome

mpMRI, multiparametric magnetic resonance imaging; no., number.

Endpoints

The primary endpoint will be statistically significant differences in quantitative measurements (for example, frequency or density) of histopathological features between mpMRI-visible and mpMRI-invisible prostate cancers. Secondary endpoints will include explanatory links between these features and mpMRI-conspicuity, as well as the potential clinical implications. As study methodology may impact estimated frequencies of histopathological features, we will include these variables in a moderator analysis (see meta-analysis below).

Risk of bias in individual studies

A modified Newcastle-Ottawa score (originally constructed for assessment of observational cohort studies) will be used to score the bias and quality across included studies.[16] This system is divided into three core elements: selection, comparability and outcome. Within each section, there are sub-questions to evaluate the quality of the research methodology, at the study level. Three reviewers will be involved with this process and

For peer review only - http://bmjopen.bmj.com/site/about/guidelines.xhtml

any disagreement will be settled by consensus. The outcome of the bias and quality assessment will inform the thematic synthesis by providing an assessment of the reliability and applicability of the available evidence. If studies are deemed to be of excessively low quality (or high bias) then these may be excluded, or if included, will be accompanied by appropriate commentary in the discussion and conclusion. As this review is focused on the histopathological characteristics associated with prostate cancer conspicuity on mpMRI (as opposed to treatment outcome), we will modify non-applicable sections of the scoring scheme to more closely reflect the nature of the evidence base and reduce reporting inaccuracy, in a similar approach that we have taken previously.[6]

Meta-analysis

If there are a sufficient number of studies available (i.e. over three) that analyse a particular histopathological feature using a similar methodology, then we will conduct a meta-analysis. The frequency of the histopathological feature between mpMRI-visible and mpMRI-invisible tumours will be compared. Any analysis will be performed as previously described.[17] Briefly, numbers of positive and negative cases of a particular histopathological feature would be extracted from a given study and raw/direct proportions will be calculated. The distribution of untransformed, logit and double arcsine transformed proportions will be compared. Whichever distributions resemble a normal distribution (assessed using density plots and Shapiro-Wilk tests) will be used for further analysis.

The model fitted will be determined based on inter-study variation (measured via I^2); if significant, a random-effects model will be fitted, or a fixed-effect model, if not. After fitting a model to all relevant studies, leave-one-out analyses (LOO) and accompanying diagnostic plots will be used to identify influential studies including: externally studentised residuals, difference in fits values (DFFITS), Cook's distances, covariance ratios, LOO estimates of the amount of heterogeneity, LOO values of the test statistics for heterogeneity, hat values and weights. Studies with a statistically significant influence on the fitted model will be removed as outliers and the model re-fitted. These outliers will be examined for potential confounding variables such as study methodology or poor inter-observer agreement for mpMRI scans. Finally, the summary estimates will be compared between mpMRI-visible and mpMRI-invisible cancers as subgroups, and the significance of differences will be assessed. If appropriate, moderator analysis will also be performed between factors which may influence the outcome, including, but not limited to: study methodology, study size, year of publication, cohort type, relevant methodology (such as radical prostatectomy versus biopsy studies) or visibility definition. Analytics will be performed as outlined by Wang.[18] In the event that there is insufficient data to conduct a meta-analysis, only thematic synthesis will be performed.

Patient and Public Involvement

No patients involved

DISCUSSION

Over the past decade, mpMRI has been widely adopted as the risk stratification tool-of-choice for men at risk of prostate cancer. This has become particularly true following incorporation of pre-biopsy mpMRI into national and international prostate cancer guidelines.[19,20] As such, it is now crucial that we better appreciate the characteristics of prostate cancers that are detected and missed by mpMRI.[4] Through systematic review, we aim to identify commonality between pathology-based studies which have investigated mpMRI-visible and mpMRI-invisible tumours. Our results will enhance the understanding of the histopathological features that influence prostate cancer visibility on mpMRI, particularly beyond increased Gleason grade and high tumour volume.

For peer review only - http://bmjopen.bmj.com/site/about/guidelines.xhtml

Estimates for proportions of significant mpMRI-undetected prostate cancer vary between studies,[21] however, it has become clear that microscopic tumour composition is likely an important factor in determining mpMRI signal (and therefore disease detection). Our planned thematic synthesis will assimilate the evidence surrounding the microscopic basis of tumour conspicuity, including the following considerations. Diffusion of water is a key principle of MRI signal generation; this is affected by alterations in tissue microenvironment and cellular structure, which may hamper free-movement of water molecules.[22] This concept theoretically explains the disparate MRI appearances of the different zones of the prostate (including, the peripheral zone and normal central gland) and is also likely to be crucial in the mpMRI-visibility of prostate cancer.[23] Indeed, an increased architectural density has been observed in mpMRI-visible tumours, with an increased proportion of cancer cells and decreased proportions of stroma and luminal spaces.[24] This may, in turn, explain visibility of these tumours on mpMRI, through increased restriction of water diffusion within dense tumour tissue (thus generating high signal on the diffusion weighted mpMRI sequence). Variations in tumour density may explain variable ability of mpMRI to estimate gross tumour volume, and may explain why alternate imaging modalities, such as PSMA-PET/CT, may provide more accurate representation of volume by targeting tumour specific markers.[25,26] It also appears that mpMRI-visible tumours have raised microvessel density,[27] which is cohesive with genetic studies that also have demonstrated an enrichment of Vascular Endothelial Growth Factor (VEGF) in these tumours.[28,29] An increased level of vasculature could potentially explain tumour visibility on mpMRI, through increased concentrations of mpMRI-contrast agent (gadolinium) in tumour vessels, thus generating higher MRI signal on the dynamic contrast sequence. Finally, there is mixed evidence regarding the mpMRI-visibility or the mpMRI-invisibility of particular prostate cancer subtypes, including cribriform pattern[30,31] and ductal cancer.[32] This is potentially concerning, as these cancer subtypes are associated with increased disease aggression and poor clinical outcome. Our planned systematic review (described in this protocol) will highlight and discuss these postulated features, among many others, in detail.

Another major aim of this review is to catalogue heterogeneity within this literature. We have recently reviewed the genetic landscape that underpins the conspicuity of prostate cancer on mpMRI and found a high degree of diversity among study methodologies, radiological scoring systems used and definitions of visibility and clinical significance.[33] This heterogeneity is further complicated by the substantial differences in scan quality observed between different centres.[34] In this proposed review, we will systematically identify sources of heterogeneity, which we hope will guide future studies in this sphere.

In summary, this systematic review will combine the extant evidence in this emerging field, for the first time. Collation and analysis of this data will enrich our understanding of the additional histopathological factors (beyond tumour grade and size) that contribute to the conspicuity of prostate cancer on mpMRI. Additionally, this process will also help reveal the potential clinical role that these factors play in both diagnosis and treatment (for example, during planning focal therapy and radiotherapy),[35,36] and will aid identification of important avenues for future research.

For peer review only

Trial status

► Preliminary searches: started.

► Piloting of the study selection process: started.

► Formal screening: started.

► Data extraction: not started.

► Risk of bias assessment: not started.

► Data analysis: not started.

Draft of search strategy for MEDLINE, EMBASE, PubMed and Cochrane Databases:

((((prostat* NOT prostatitis) AND ("cancer" OR tumo?r* OR malignancy*)) AND ("magnetic resonance imaging" OR "MRI" OR "multiparametric magnetic resonance imaging" OR "mpMRI" OR "mp-MRI")) AND (patholog* OR histopatholog* OR histo* OR "IHC" OR "Immunohistochemistry")) AND visib*).ti,ab

ETHICS AND DISSEMINATION

Due to the nature of the study, there are no relevant ethical concerns and informed consent will not be required. The protocol and systematic review will be disseminated via a peer-reviewed journal.

Author affiliations

[1] UCL Division of Surgery & Interventional Science, University College London, London, UK

[2] London Deanery of Urology, London, UK

[3] Department of Urology, University College London Hospitals NHS Foundation Trust, London, UK

[4] Department of Radiology, University College London Hospitals NHS Foundation Trust, London, UK

[5] Department of Pathology, University College London Hospitals NHS Foundation Trust, London, UK

[6] School of Healthcare Sciences, Cardiff University, Wales, UK

Twitter Joseph M Norris @MrJosephNorris, Lina M Carmona Echeverria @linamacarmona, Benjamin S Simpson @b3nsimp, Daniel Kelly @profdkelly, Hayley C Whitaker @HayleyWhitaker, Mark Emberton @EmbertonMark

Contributors The authors' contribution includes, but is not limited to, the following: JMN, LMCE and BSS drafted the manuscript and created the study concept. RB, AK, AF, DK, HCW and ME provided supervision and guidance during the study. All authors reviewed and approved the manuscript in its current form.

Funding JMN is funded by the Medical Research Council (MRC) (MR/S00680X/1). LMCE receives funding from Prostate Cancer UK (PCUK). BSS receives funding from the Rosetrees Trust.

Competing interests Norris receives funding from the MRC. Simpson receives funding from the Rosetrees Trust. Carmona Echeverria receives funding from PCUK. Whitaker receives funding from PCUK, the Urology Foundation and the Rosetrees Trust. Kirkham, Freeman and Emberton

For peer review only

have stock interest in Nuada Medical Ltd. Emberton receives funding from NIHR-i4i, MRC, Sonacare Inc., Trod Medical, Cancer Vaccine Institute and Sophiris Biocorp for trials in prostate cancer. Emberton is a medical consultant to Sonacare Inc., Sophiris Biocorp, Steba Biotech, GSK, Exact Imaging and Profound Medical. Travel allowance was previously provided from Sanofi Aventis, Astellas, GSK and Sonacare. Emberton is a proctor for HIFU with Sonacare Inc. and paid for training other surgeons in this procedure.

Patient consent for publication Not required.

Provenance and peer review Not commissioned; externally peer reviewed.

Open access This is an open access article distributed in accordance with the Creative Commons Attribution 4.0 Unported (CC BY 4.0) license, which permits others to copy, redistribute, remix, transform and build upon this work for any purpose, provided the original work is properly cited, a link to the licence is given, and indication of whether changes were made. See: https://creativecommons.org/licenses/by/4.0/.

ORCID IDs

Joseph M Norris http://orcid.org/0000-0003-2294-0303

Benjamin S Simpson http://orcid.org/0000-0003-3685-6110

For peer review only

REFERENCES

1. Ahmed HU, El-Shater Bosaily A, Brown LC, *et al*. Diagnostic accuracy of multi-parametric MRI and TRUS biopsy in prostate cancer (PROMIS): a paired validating confirmatory study. *Lancet* 2017;389:815–22.
2. Kasivisvanathan V, Rannikko AS, Borghi M, *et al*. MRI-targeted or standard biopsy for prostate-cancer diagnosis. *N Engl J Med* 2018;378:1767–77.
3. Radtke JP, Kuru TH, Boxler S, *et al*. Comparative analysis of transperineal template saturation prostate biopsy versus magnetic resonance imaging targeted biopsy with magnetic resonance imaging-ultrasound fusion guidance. *J Urol* 2015;193:87–94.
4. Norris JM, Carmona Echeverria LM, Bott SRJ, *et al.* Which prostate cancers are overlooked by mpMRI? An analysis from PROMIS. *J Urol* 2020;203:e1243–4.
5. Norris JM, Simpson BS, Parry MA, *et al*. Genetic correlates of prostate cancer visibility (and invisibility) on mpMRI: it's time to take stock. *BJU Int* 2020;125:340–2.
6. Norris JM, Simpson, BS, Parry MA, *et al*. Genetic landscape of prostate cancer conspicuity on multiparametric MRI: a protocol for a systematic review and bioinformatic analysis. *BMJ Open* 2020;10:e03461.
7. Bill-Axelson A, Holmberg L, Garmo H, *et al*. Radical prostatectomy or watchful waiting in prostate cancer – 29-year follow-up. *NEJM* 2018;379:2319–29.
8. Kir G, Sarbay BC, Gümüş E, *et al*. The association of the cribriform pattern with outcome for prostatic adenocarcinomas. *Pathol Res Pract* 2014;210:640–4.
9. Kweldam CF, Wildhagen MF, Steyerberg EW, *et al*. Cribriform growth is highly predictive for postoperative metastasis and disease-specific death in Gleason score 7 prostate cancer. *Mod Pathol* 2015;28:457–64.
10. Khani F, Epstein JI. Prostate biopsy specimens with Gleason 3+3=6 and intraductal carcinoma. *Am J Surg Pathol* 2015;39:1383–9.
11. Mucci LA, Powolny A, Giovannucci E, *et al*. Prospective study of prostate tumor angiogenesis and cancer-specific mortality in the health professionals follow-up study. *J Clin Oncol* 2009;27:5627–33.
12. Deniffel D, Salinas E, Ientilucci M, *et al.* Does the visibility of grade group 1 prostate cancer on baseline multiparametric magnetic resonance imaging impact clinical outcomes? *J Urol* 2020;101097JU0000000000001157.
13. Ahdoot M, Wilbur, AR, Reese SE, *et al*. MRI-targeted, systematic, and combined biopsy for prostate cancer diagnosis. *N Eng J Med* 2020;382:917–28.
14. Moher D, Shamseer L, Clarke M, *et al*. Preferred reporting items for systematic review and meta-analysis protocols (PRISMA-P) 2015 statement. *Syst Rev* 2015;4:1.
15. Wells G, Shea B, Oconnell D. The Newcastle-Ottawa scale (NOS) for assessing the quality of nonrandomized studies in meta-analyses. Available from: ohri.ca/programs/clinical_epidemiology/oxford.asp
16. Ouzzani M, Hammady H, Fedorowicz Z, *et al*. Rayyan—a web and mobile app for systematic reviews. *Syst Rev* 2016 Dec;5(1):210.
17. O'Connor E, Simpson BS, Houlden H, *et al*. Prevalence of familial cluster headache: a systematic review and meta-analysis. *J Headache Pain* 2020;21:37.
18. Wang N. How to conduct a meta-analysis of proportions in R: a comprehensive tutorial. DOI: 10.13140/RG.2.2.27199.00161.
19. Wise J. NICE recommends MRI for suspected prostate cancer to reduce biopsies. BMJ 2018;363:k5290.
20. Dasgupta P, Davis J, Hughes S. NICE guidelines on prostate cancer 2019. *BJU Int* 2019;124:1.

For peer review only - http://bmjopen.bmj.com/site/about/guidelines.xhtml

21. Le JD, Tan N, Shkolyar E, *et al*. Multifocality and prostate cancer detection by multiparametric magnetic resonance imaging: correlation with whole-mount histopathology. *Eur Urol* 2015;67:569–76.

22. Le Bihan D. Molecular diffusion nuclear magnetic resonance imaging. *Magn Reson Q* 1991;7:1–30.

23. Kozlowski P, Chang SD, Jones EC, *et al*. Combined diffusion-weighted and dynamic contrast-enhanced MRI for prostate cancer diagnosis—correlation with biopsy and histopathology. *J Magn Reson Imaging* 2006;24:108–13.

24. Miyai K, Mikoshi A, Hamabe F, *et al*. Histological differences in cancer cells, stroma, and luminal spaces strongly correlate with in vivo MRI-detectability of prostate cancer. *Mod Pathol* 2019;32:1536–43.

25. Bettermann AS, Zamboglou C, Kiefer S, *et al*. [68Ga-] PSMA-11 PET/CT and multiparametric MRI for gross tumor volume delineation in a slice by slice analysis with whole mount histopathology as a reference standard–Implications for focal radiotherapy planning in primary prostate cancer. *Radiother Oncol* 2019;141:214–9.

26. Zamboglou C, Drendel V, Jilg CA, *et al*. Comparison of 68Ga-HBED-CC PSMA-PET/CT and multiparametric MRI for gross tumour volume detection in patients with primary prostate cancer based on slice by slice comparison with histopathology. *Theranostics* 2017;7:228–37.

27. Borren A, Groenendaal G, Moman MR, *et al*. Accurate prostate tumour detection with multiparametric magnetic resonance imaging: dependence on histological properties. *Acta Oncol* 2014;53:88–95.

28. Lenkinski RE, Bloch BN, Liu F, *et al*. Mapping MRI/MRS parameters with genetic over-expression profiles in human prostate cancer: demonstrating the potential. *MAGMA* 2008;21:411–21.

29. Li P, You S, Nguyen C, *et al*. Genes involved in prostate cancer progression determine MRI visibility. *Theranostics* 2018;8:1752–65.

30. Tonttila PP, Ahtikoski A, Kuisma M, *et al*. Multiparametric MRI prior to radical prostatectomy identifies intraductal and cribriform growth patterns in prostate cancer. *BJU Int* 2019;124:992–99.

31. Truong M, Feng C, Hollenberg G, *et al*. A comprehensive analysis of cribriform morphology on magnetic resonance imaging/ultrasound fusion biopsy correlated with radical prostatectomy specimens. *J Urol* 2018;199:106–13.

32. Schieda N, Coffey N, Gulavita P, *et al*. Prostatic ductal adenocarcinoma: an aggressive tumour variant unrecognized on T2 weighted magnetic resonance imaging (MRI). *Eur Radiol* 2014;24:1349–56.

33. Norris JM, Simpson BS, Parry MA, *et al*. Genetic landscape of prostate cancer conspicuity on multiparametric MRI: a systematic review and bioinformatic analysis. *Eur Urol Open Sci* 2020;20;37–47.

34. Sonn GA, Fan RE, Ghanouni P, *et al.* Prostate magnetic resonance imaging interpretation varies substantially across radiologists. *European Urol Focus* 2019;5:592–9.

35. Scheltema MJ, Tay KJ, Postema AW. Utilization of multiparametric prostate magnetic resonance imaging in clinical practice and focal therapy: report from a Delphi consensus project. *World J Urol* 2017;35:695–701.

36. Zamboglou C, Klein CM, Thomann B, *et al.* The dose distribution in dominant intraprostatic tumour lesions defined by multiparametric MRI and PSMA PET/CT correlates with the outcome in patients treated with primary radiation therapy for prostate cancer. *Radiat Oncol* 2018;13:65.

For peer review only

PRISMA-P (Preferred Reporting Items for Systematic review and Meta-Analysis Protocols) 2015 checklist: recommended items to address in a systematic review protocol*

	Section and topic	Item No	Checklist item
	**ADMINISTRATIVE INFORMATION**		
	Title:		
✓ Page 1	Identification	1a	Identify the report as a protocol of a systematic review
✗ N/A	Update	1b	If the protocol is for an update of a previous systematic review, identify as such
✗ See cover letter	Registration	2	If registered, provide the name of the registry (such as PROSPERO) and registration number
	Authors:		
✓ Page 1	Contact	3a	Provide name, institutional affiliation, e-mail address of all protocol authors; provide physical mailing address of corresponding author
✓ Page 8	Contributions	3b	Describe contributions of protocol authors and identify the guarantor of the review
✗ N/A	Amendments	4	If the protocol represents an amendment of a previously completed or published protocol, identify as such and list changes; otherwise, state plan for documenting important protocol amendments
	Support:		
✓ Page 8	Sources	5a	Indicate sources of financial or other support for the review
✓ Page 8	Sponsor	5b	Provide name for the review funder and/or sponsor
✓ Page 8	Role of sponsor or funder	5c	Describe roles of funder(s), sponsor(s), and/or institution(s), if any, in developing the protocol
	**INTRODUCTION**		
✓ Page 3	Rationale	6	Describe the rationale for the review in the context of what is already known
✓ Page 5	Objectives	7	Provide an explicit statement of the question(s) the review will address with reference to participants, interventions, comparators, and outcomes (PICO)
	**METHODS**		
✓ Page 4	Eligibility criteria	8	Specify the study characteristics (such as PICO, study design, setting, time frame) and report characteristics (such as years considered, language, publication status) to be used as criteria for eligibility for the review
✓ Page 4	Information sources	9	Describe all intended information sources (such as electronic databases, contact with study authors, trial registers or other grey literature sources) with planned dates of coverage
✓ Page 8	Search strategy	10	Present draft of search strategy to be used for at least one electronic database, including planned limits, such that it could be repeated
	Study records:		
✓ Page 4/5	Data management	11a	Describe the mechanism(s) that will be used to manage records and data throughout the review

For peer review only

✓ Page 4	Selection process	11b	State the process that will be used for selecting studies (such as two independent reviewers) through each phase of the review (that is, screening, eligibility and inclusion in meta-analysis)
✓ Page 5	Data collection process	11c	Describe planned method of extracting data from reports (such as piloting forms, done independently, in duplicate), any processes for obtaining and confirming data from investigators
✓ Page 5	Data items	12	List and define all variables for which data will be sought (such as PICO items, funding sources), any pre-planned data assumptions and simplifications
✓ Page 5	Outcomes and prioritization	13	List and define all outcomes for which data will be sought, including prioritization of main and additional outcomes, with rationale
✓ Page 5	Risk of bias in individual studies	14	Describe anticipated methods for assessing risk of bias of individual studies, including whether this will be done at the outcome or study level, or both; state how this information will be used in data synthesis
✓ Page 6	Data synthesis	15a	Describe criteria under which study data will be quantitatively synthesised
		15b	If data are appropriate for quantitative synthesis, describe planned summary measures, methods of handling data and methods of combining data from studies, including any planned exploration of consistency (such as I^2, Kendall's τ)
		15c	Describe any proposed additional analyses (such as sensitivity or subgroup analyses, meta-regression)
		15d	If quantitative synthesis is not appropriate, describe the type of summary planned
✓ Page 5	Meta-bias(es)	16	Specify any planned assessment of meta-bias(es) (such as publication bias across studies, selective reporting within studies)
✓ Page 5	Confidence in cumulative evidence	17	Describe how the strength of the body of evidence will be assessed (such as GRADE)

*** It is strongly recommended that this checklist be read in conjunction with the PRISMA-P Explanation and Elaboration (cite when available) for important clarification on the items. Amendments to a review protocol should be tracked and dated. The copyright for PRISMA-P (including checklist) is held by the PRISMA-P Group and is distributed under a Creative Commons Attribution Licence 4.0.**

From: Shamseer L, Moher D, Clarke M, Ghersi D, Liberati A, Petticrew M, Shekelle P, Stewart L, PRISMA-P Group. Preferred reporting items for systematic review and meta-analysis protocols (PRISMA-P) 2015: elaboration and explanation. BMJ. 2015 Jan 2;349(jan02 1):g7647.

For peer review only
